# Supplementary material for: Selective self-assembly of 2,3-diaminophenazine molecules on MoSe2 mirror twin boundaries
Source: Nat Commun. 2019 Jun 28;10:2847. doi: 10.1038/s41467-019-10801-0 (PMC6599086; doi:10.1038/s41467-019-10801-0)
Supplement: Supplementary file 1 — Supplementary Information [file 41467_2019_10801_MOESM1_ESM.pdf]

Supplementary Information

Selective self-assembly of 2,3-diaminophenazine molecules on $MoSe_2$ mirror twin boundaries

Xiaoyue He[1], Lei Zhang[1], Rebekah Chua[1,3], Ping Kwan Johnny Wong[4], Arramel[1], Yuan Ping Feng[1], Shi Jie Wang[2], Dongzhi Chi[2], Ming Yang[2], Yu Li Huang[1,2] and Andrew Thye Shen Wee[1,4]

[1]Department of Physics, National University of Singapore, 2 Science Drive 3, Singapore 117542, Singapore

[2]Institute of Materials Research & Engineering (IMRE), A*STAR (Agency for Science, Technology and Research), 2 Fusionopolis Way, Innovis, Singapore 138634, Singapore

[3]NUS Graduate School for Integrative Sciences & Engineering (NGS), National University of Singapore, 28 Medical Drive, Singapore 117456, Singapore

[4]Centre for Advanced 2D Materials (CA2DM) and Graphene Research Centre (GRC), National University of Singapore, 117546 Singapore

Correspondence and requests for materials should be addressed to M.Y. (email: yangm@imre.a-star.edu.sg) or to Y.L.H. (email:chocosea@gmail.com) or to A.T.S.W. (email: phyweets@nus.edu.sg).

The electronic properties of DAP molecule.

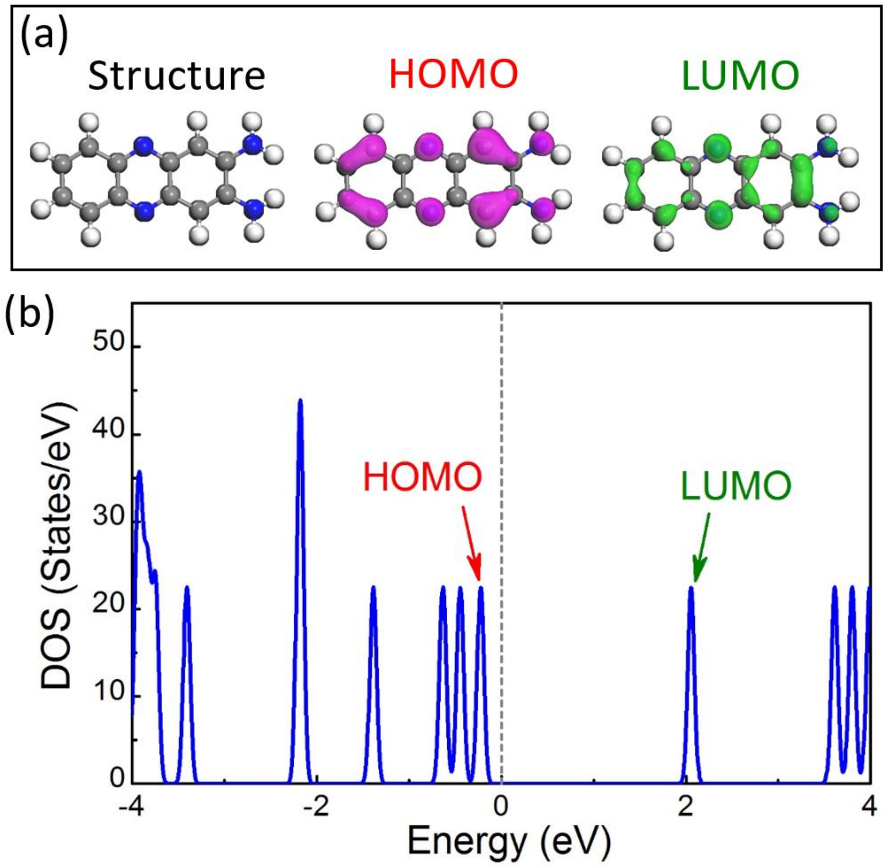

Supplementary Figure 1: a, The schematic of DAP molecule structure and visualized HOMO, LUMO. **b,** Density state of DAP molecule.

Large scale STM image of DAP porous structure.

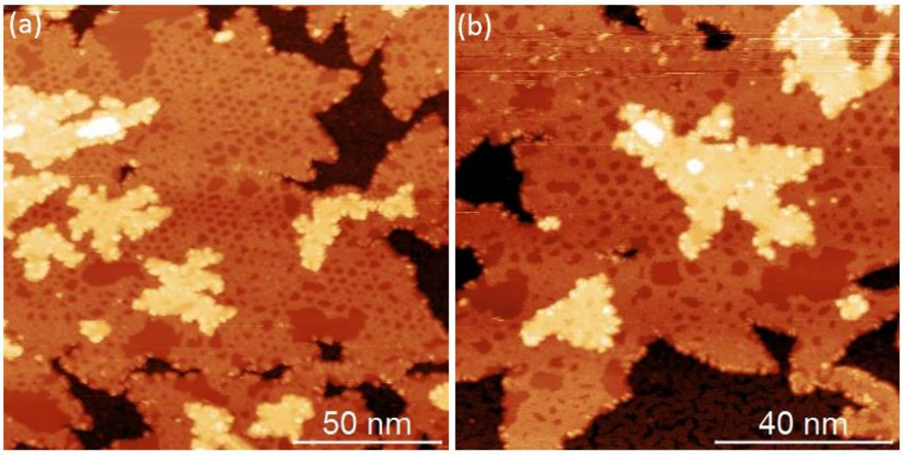

Supplementary Figure 2: Large scale STM image of 0.8 ML DAP molecule on $MoSe_2$. **a,** 150 × 150 nm^2 (2.3 V, 90 pA); **b,** 100 × 100 nm^2 (2.6 V, 60 pA). It shows that this DAP pores can extend over the $MoSe_2$ flakes.

The stability of DAP molecules absorbed on $MoSe_2$.

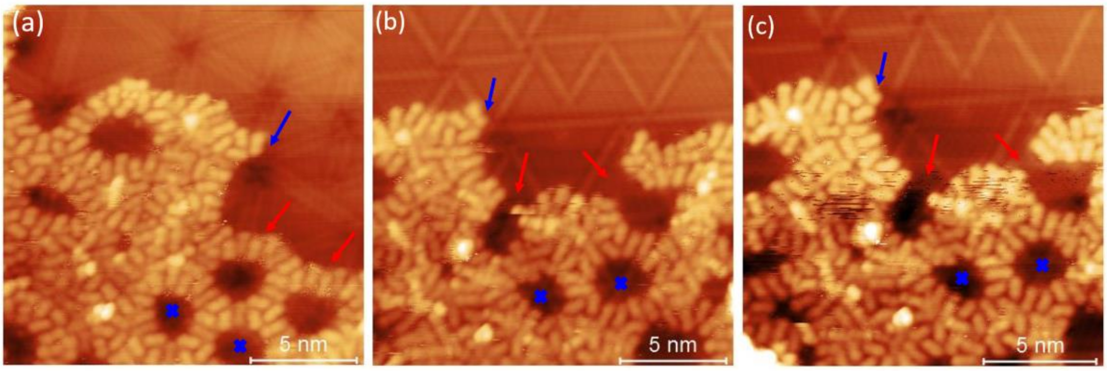

Supplementary Figure 3: a-c, Rescanned times (n) dependent STM images of DAP porous structure at the area (2.0 V, 100 pA) with subsequence n = 1, 4 and 7, respectively. The blue arrows mark the same DAP molecule staying steadily during the scanning. The red arrows show the area of DAP molecules moving during scanning. It indicates that the adsorption of DAP molecules on the $MoSe_2$ surface is relatively weak.

Statistical analysis of the orientation distribution of DAP on MTB region.

Supplementary Figure 4a is the schematic of the definition of the rotation angle (θ) of DAP relative to MTBs, where the grey arrows highlight the MTB orientations, and the blue arrow lines mark the long axis of DAP molecules. The MTBs are three-fold symmetric and have three equivalent directions, and the DAP adsorption positions (i.e., the molecular centre) are usually centred away from the MTBs (see Figure 4b and d). For this statistical analysis, we only consider the orientation of the selected molecule relative to one selected MTB orientation. To be consistent with the theoretical model, the statistical step is set to 10° (e.g., 0-10°, 10-20°, etc.). From the histogram, we can see that the molecules orientated at ~0° are dominant (~30.8%), compared to the other orientations. This result agrees with the calculated angle-dependence adsorption energy of DAP molecule adsorbed on MTB shown in Figure 6, which suggests that the molecule adsorbed parallel (0°) to the MTB with lowest binding energy. The relatively weak preference agrees with the fact that the calculated binding energy difference to the other orientations are not large, i.e., < 0.16 eV.

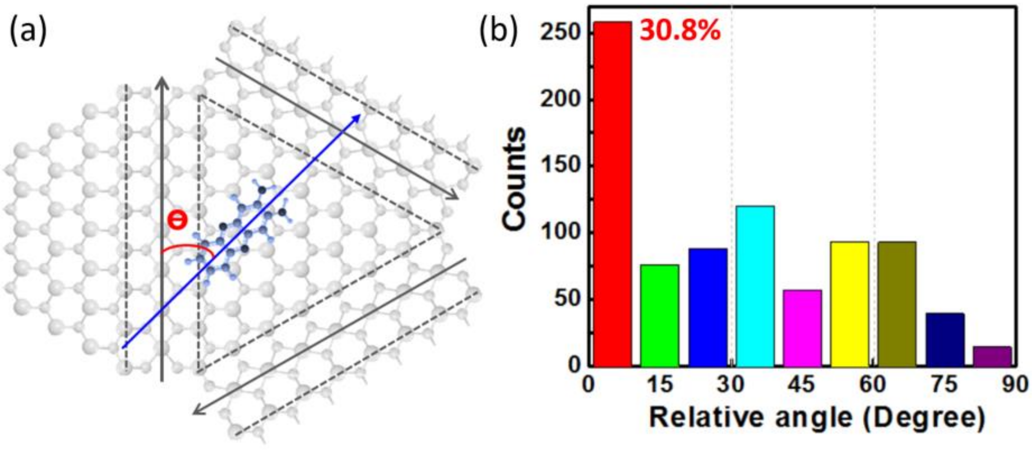

Supplementary Figure 4: a, The schematic of the rotation angle (ɵ) of DAP relative to MTBs. The grey arrows show the directions of MTBs; the blue arrow shows the long axis of DAP molecules. **b**, A statistical analysis of the orientation distribution of DAP (more than 800 DPA molecules) relative to MTBs. From the histogram, we can see that the molecules orientated at ~0° are dominant (~30.8%), compared to other orientations.

Statistical analysis of the distribution of the two types (L-type and T-type) of configuration of DAPs.

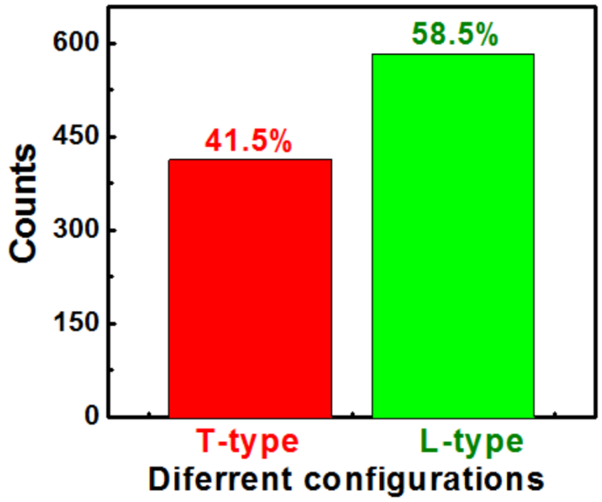

Supplementary Figure 5: A statistical analysis of the distribution of the two types of configuration of DAPs (~1000 DAP molecules). It is found that the percentage of L-type configurations, 58.5%, is higher than that of T-type one. This observation is in contrast to the relatively higher formation energy per molecule for the T-type configuration (-0.21 eV) than the L-type (-0.12 eV) without considering their adsorption sites. As the L-type configurations are preferentially adsorbed atop the MTBs while the T-types likely to the $MoSe_2$ domains, the slightly preferential L-type configuration is resulted in by stronger interactions between the DAP molecules and the MTBs.

High coverage of DAP molecules.

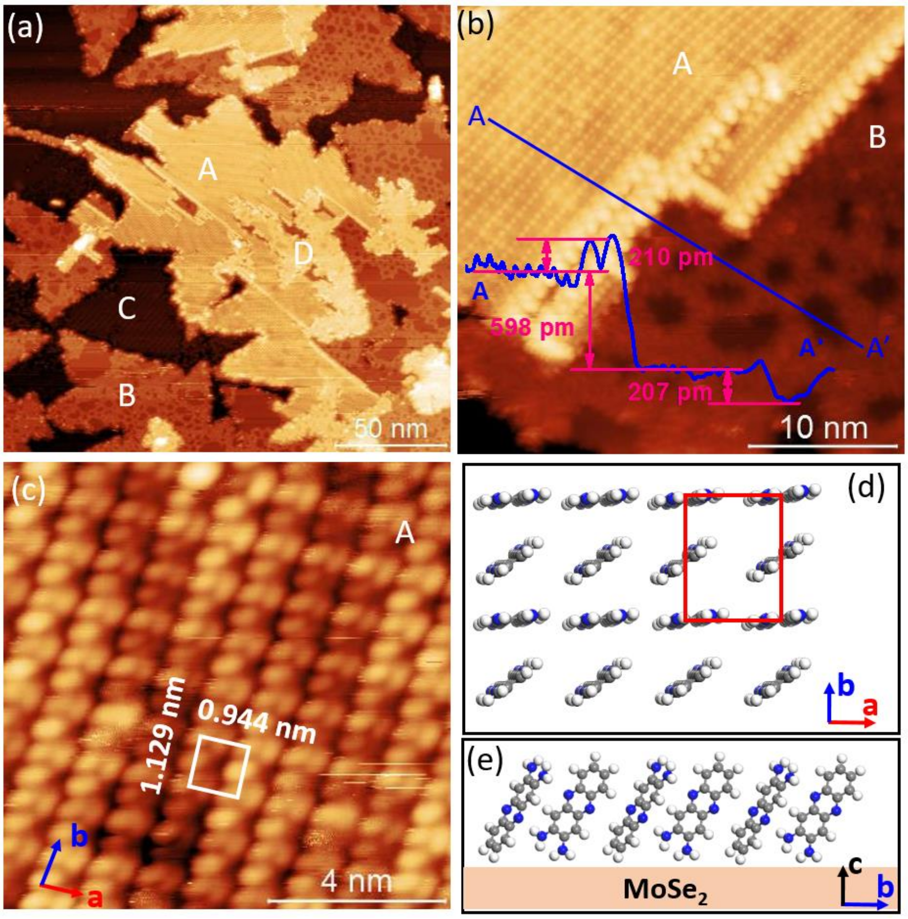

Supplementary Figure 6: a, Large-scale STM image (2.3 V, 90 pA; 200 × 200 nm^2) of 1.2 ML DAP on $MoSe_2$. Region A, B, C and D highlight the close packing monolayer DAP on monolayer $MoSe_2$, sub-monolayer DAP porous structure on monolayer $MoSe_2$, monolayer DAP on HOPG and sub-monolayer DAP on the bilayer $MoSe_2$ islands, respectively. **b,** Magnified STM image (2.3 V, 70 pA; 25 × 25 nm^2) of the region A and B. The insert is the line-profile corresponding to the blue line AA'. It shows that the height of sub-monolayer DAP porous structure is about 207 pm, while the close-packing DAP monolayer is about 805 pm. **c,** High resolution STM (2.0 V, 70 pA; 10 × 10 nm^2) image of region A. The unit cell is highlighted by a white rectangle, with a = 0.944 ± 0.05 nm and b = 1.129 ± 0.035 nm. **d-e,** Schematic models of the inclined packing structure of monolayer DAP on $MoSe_2$ in region A.

The influence of annealing temperature.

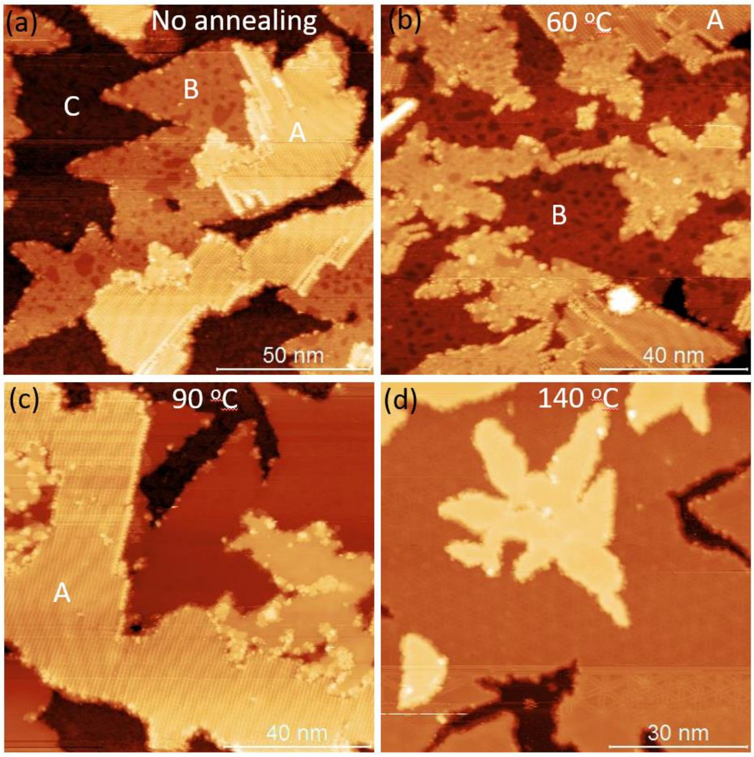

Supplementary Figure 7: a, STM image (2.3 V, 70 pA; 120 × 120 nm^2) of 1.1 ML DAP molecule on the $MoSe_2$ surface before annealing. **b-d,** The DAP samples after annealing at 60°C, 90°C, and 140°C respectively. Region A, B and C mark the close-packing DAP on $MoSe_2$, porous DAP monolayer on $MoSe_2$ and monolayer DAP on HOPG. In panel b, both the porous DAP structures and the close-packing structures are well resolved after annealing at 60°C. In panel c, the porous structures disappear while the close-packing structures remain upon annealing at 90°C. In panel d, the close-packed DAP monolayer also desorb leaving the $MoSe_2$ empty after annealing at 140°C. (b, 2.5 V, 60 pA, 100 × 100 nm^2; c, 2.5 V, 60 pA; 100 × 100 nm^2; d, -2.6 V, 80 pA; 80 × 80 nm^2).

DAP molecule on HOPG substrate.

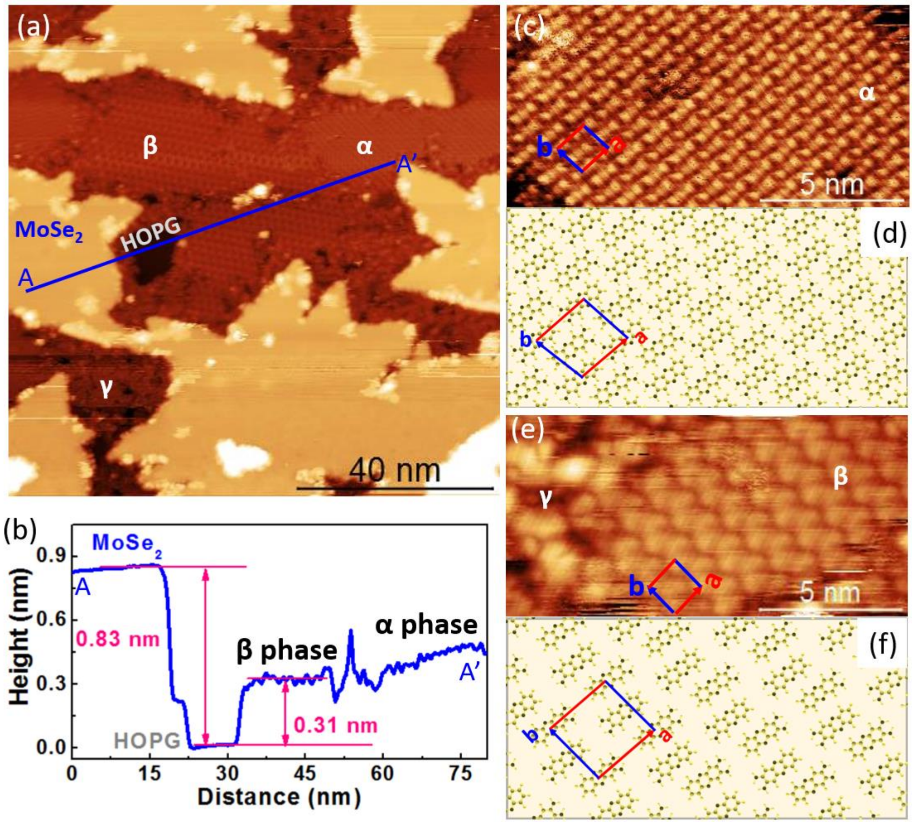

Supplementary Figure 8: a, Large area STM image (2.7 V, 60 pA; 100 × 100 nm^2) of monolayer DAP on HOPG. α, β and γ phase show the dense close-packing, spare close-packing and disorder structure. **b,** The line-profile corresponding to the blue line AA' in panel a. The height of monolayer DAP molecules on HOGP is about 310 pm. The height of α and β phase almost are same, indicating that both of them are flat lying on the HOPG substrate. **c-d,** Magnified STM image **(**2.7 V, 60 pA; 14 × 7 nm^2) and schematic diagram of α phase. Here, a = 1.24 ± 0.05 nm, b = 1.17 ± 0.05 nm. **e-f,** Magnified STM image **(**2.3 V, 60 pA; 14 × 7 nm^2) and schematic diagram of β phase. Here, a = 1.249 ± 0.05 nm, b = 1.076 ± 0.05 nm. It shows that the DAP molecules on graphite assemble into close-packed ordered arrays.

DAP molecules on Au.

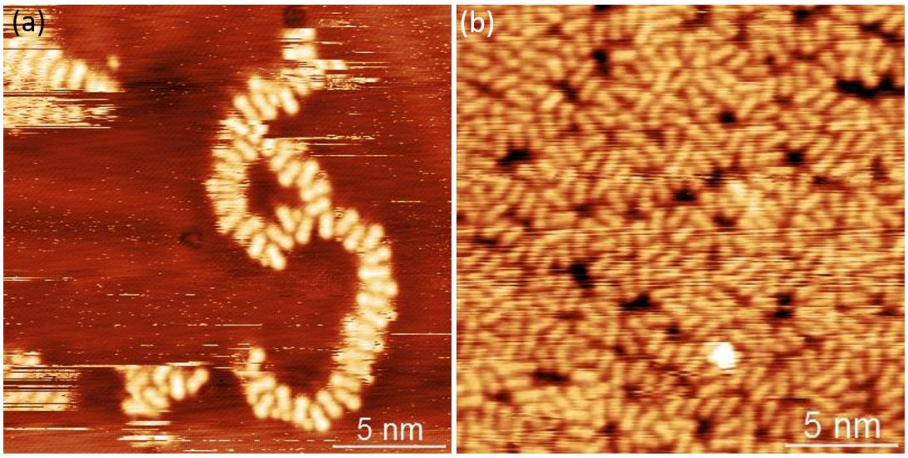

Supplementary Figure 9: DAP molecules on Au(111). **a,** STM image (-0.98 V, 60 pA; 20 × 20 nm^2) of DAP molecules on Au at low coverage. It shows a chain-like structure due to intermolecular hydrogen bonding. **b,** STM image (-1.06 V, 60 pA; 20 × 20 nm^2) of monolayer DAP on Au(111) shows irregular packing structure. It shows that the DAP molecules on Au assemble into chain-like structures at low coverage and a disordered packing structure at higher coverage

The optimized aggregated configuration of DAP T-type and L-type.

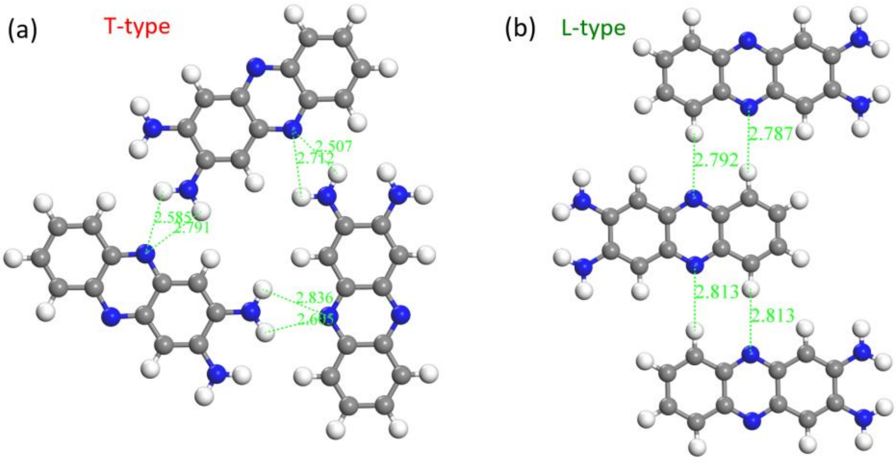

Supplementary Figure 10: a-b, The hydrogen bonds of T-type and L-type aggregation configuration of DAP without substrate, respectively. The C-N···H distance between the neighbouring DAP molecules is in the range of 2.5 - 2.9 Å, indicating weak hydrogen-bonding.

Atomic configuration of the DAP adsorbed on D site, P site and V site.

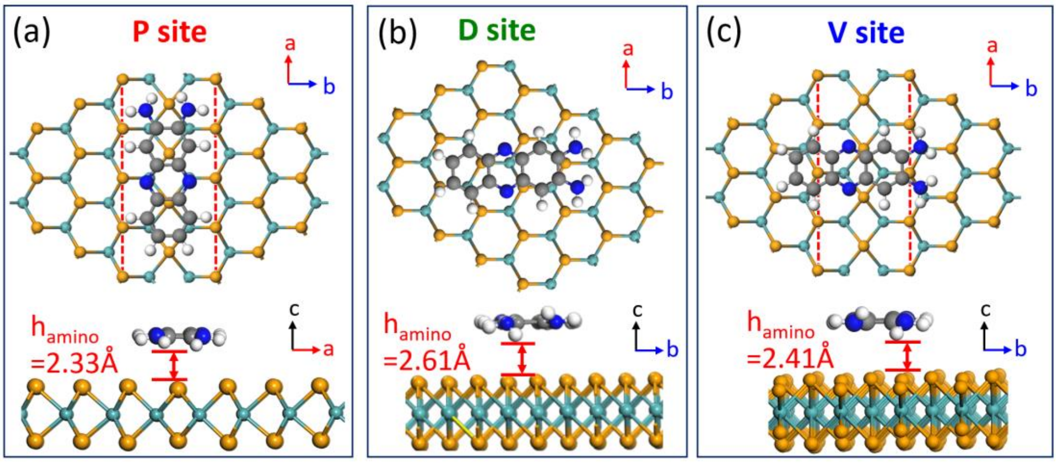

Supplementary Figure 11: a-c, Top view and side view of DAP adsorbed on MTBs with P site, D site and V site. h_{amino} is the equilibrium distance from the amino group to the top-layer Se of $MoSe_2$ surface. It is clear that the orientation of hydrogens on two amino group is opposite for D site and V site, while both of them are bend to the $MoSe_2$ surface on P site due to the slightly strong interaction with MTBs.

The optimized configuration of DAP adsorbed on V site.

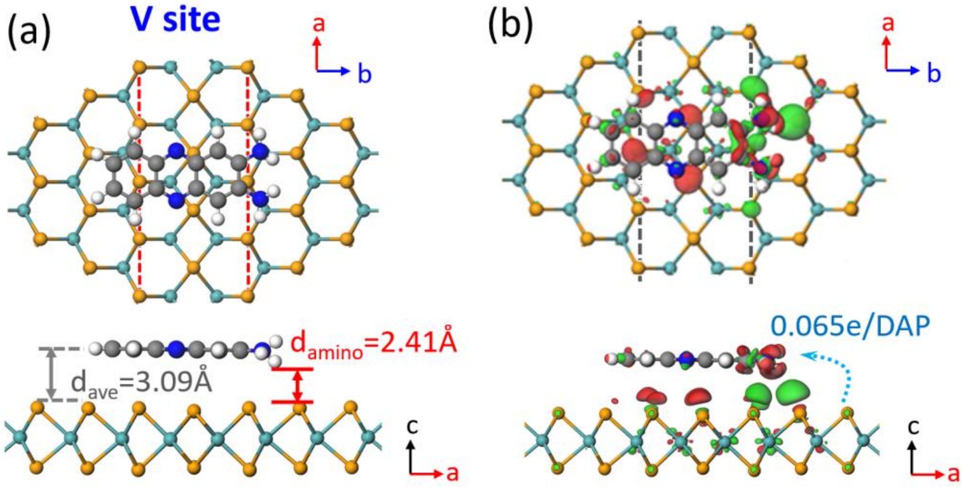

Supplementary Figure 12: a, Top view and side view of DAP adsorbed on MTBs with a perpendicular orientation to the MTB, named V site. h_{ave} and h_{amino} are the equilibrium distance from the center of DAP molecule or amino group to the top-layer Se of $MoSe_2$ surface. **b,** Charge density difference of P site and D site calculated by $\boldsymbol{\rho} = \boldsymbol{\rho}_{\mathbf{MoSe2+DAP}} - \boldsymbol{\rho}_{\mathbf{MoSe2}} - \boldsymbol{\rho}_{\mathbf{DAP}}$. The red and green distributions denote the electron accumulation and depletion regions, respectively. The isosurface value of the charge density was set to be 4×1.0^{-4} e/Å^3. The blue dash-line shows the charge transfer from $MoSe_2$ to DAP molecule calculated by integrating the in-plane averaged charge density difference.

Charge density difference.

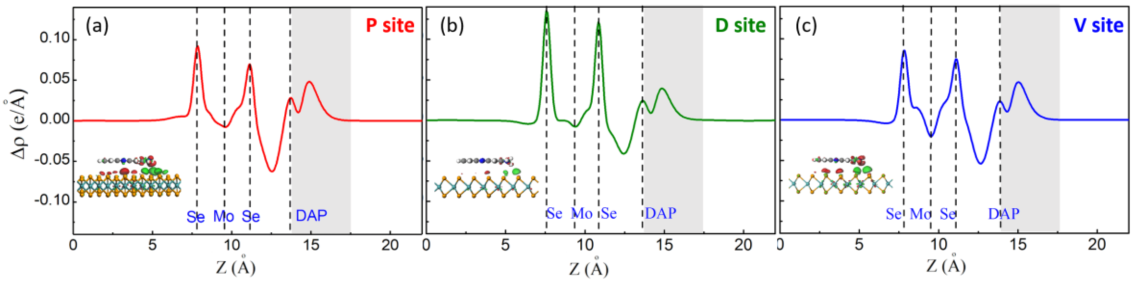

Supplementary Figure 13: a-c, The in-plane averaged of $\Delta\rho(z)$ between DAP and $MoSe_2$ at D site, P site and V site, respectively. The inset is their corresponding side view of charge density difference. The isosurface value of the charge density difference was set to be 4×1.0^{-4} e/Å^3.

Summary of calculated results for different DAP adsorbed position.

Adsorbed position	E_{ads} (eV)	h_{ave} (Å)	h_{amino} (Å)	CT (e/Mo) by Bader	CT(e/Mo) by Integrated	Schematic
**D site**	-1.383	2.98	2.33	0.05	0.07	Fig. 5(c)
**P site**	-1.22	3.22	2.61	0.03	0.05	Fig. 5(e)
**V site**	-1.29	3.09	2.41	0.04	0.065	Fig. S4

Supplementary Table 1. Calculated results for DAP aggregation binding energy and isolated DAP molecules on monolayer $MoSe_2$ with three different configurations: adsorption energy (E_{ads}), equilibrium height between the center of DAP molecule (h_{ave})/amino group (h_{amino}) and the top Se-layer of the $MoSe_2$ sheet, amounts of charge transferred (CT) from the $MoSe_2$ monolayer into DAP molecule obtained from the integration of in-plane averaged charge density difference.
